# Development of a PCR Assay to Detect Low Level *Trypanosoma cruzi* in Blood Specimens Collected with PAXgene Blood DNA Tubes for Clinical Trials Treating Chagas Disease

**DOI:** 10.1371/journal.pntd.0005146

**Published:** 2016-12-01

**Authors:** Bo Wei, Lei Chen, Miho Kibukawa, John Kang, Hetty Waskin, Matthew Marton

**Affiliations:** 1 Merck Research Laboratories, Translational Molecular Biomarkers, Rahway, New Jersey, United States of America; 2 Merck Research Laboratories, Biometrics Research, Rahway, New Jersey, United States of America; 3 Merck Research Laboratories, Infectious Disease, Rahway, New Jersey, United States of America; 4 Merck Research Laboratories, Companion Diagnostics, Rahway, New Jersey, United States of America; US Food and Drug Administration, UNITED STATES

## Abstract

Chagas disease is caused by the parasitic infection of *Trypanosoma cruzi* (*T*. *cruzi*). The STOP CHAGAS clinical trial was initiated in 2011 to evaluate posaconazole in treating Chagas disease, with treatment success defined as negative qualitative PCR results of detecting the parasites in blood specimens collected post-treatment. PAXgene Blood DNA tubes were utilized as a simple procedure to collect and process blood specimens. However, the PAXgene blood specimens challenged published *T*. *cruzi* PCR methods, resulting in poor sensitivity and reproducibility. To accurately evaluate the treatment efficacy of the clinical study, we developed and validated a robust PCR assay for detecting low level *T*. *cruzi* in PAXgene blood specimens. The assay combines a new DNA extraction method with a custom designed qPCR assay, resulting in limit of detection of 0.005 and 0.01 fg/μl for K98 and CL Brener, two representative strains of two of *T*. *cruzi*’s discrete typing units. Reliable qPCR standard curves were established for both strains to measure parasite loads, with amplification efficiency ≥ 90% and the lower limit of linearity ≥ 0.05 fg/μl. The assay successfully analyzed the samples collected from the STOP CHAGAS study and may prove useful for future global clinical trials evaluating new therapies for asymptomatic chronic Chagas disease.

## Author Summary

Chagas disease is caused by the infection of the protozoan parasite *Trypanosoma cruzi* (*T*. *cruzi*) and carries a significant tropical disease burden in the Western Hemisphere. The STOP CHAGAS study was a global clinical trial evaluating therapies for Chagas disease. PAXgene blood DNA tubes used by the study simplified and standardized the sample collection and processing procedures, but challenged published PCR assays that detect circulating *T*. *cruzi*. In this study, we report our effort of developing and validating a robust, sensitive and specific PCR assay for detecting *T*. *cruzi* in PAXgene blood specimens. The efficacy outcome results of the STOP CHAGAS study that utilized our assay further support the use of the PCR assay as a marker of therapeutic response for patients with Chagas disease.

## Introduction

Chagas disease (CD) is a tropical parasitic disease caused by the chronic infection with the protozoan *T*. *cruzi*. About 6 to 7 million people, primarily in Mexico and Central and South America, are estimated to be infected [1]. Left untreated, up to 30 to 40% of chronically infected patients will progress to have symptomatic disease, manifested as serious heart and digestive problems [2], with an estimate of more than 10,000 deaths annually [3]. Current anti-parasitic medications such as benzindazole (BNZ), although highly effective in treating acute phase infection, have limited efficacy in eliminating *T*. *cruzi* for chronically infected patients and also can induce severe side effects [4]. The STOP CHAGAS study [5] (ClinicalTrials.gov Identifier NCT01377480) was a Phase 2 proof-of-activity clinical trial comparing posaconazole (POS) with BNZ in treating patients with asymptomatic chronic CD. It was a randomized placebo-controlled study conducted in nineteen centers in five Latin American countries and Spain. The study’s primary end point was qualitative PCR results of detecting *T*. *cruzi* in whole blood samples collected post-treatment, with the treatment success response defined as the consecutive negative qualitative PCR results of blood collected on at least three post-treatment dates. The reason why persistent negative PCR results of multiple time points were utilized to define treatment success was due to low levels of circulating parasites in chronic CD patients [6]. To minimize false negative PCR results and thus reduce the false positive efficacy outcome, a PCR assay with high sensitivity and specificity was required. The PCR assay was initially performed by a contract research organization (CRO) that used a widely used commercial kit to extract DNA from a 0.2 ml aliquot of PAXgene blood, and a published TaqMan based qPCR assay targeting minicircle DNA of *T*. *cruzi*’s kinetoplast DNA (kDNA) [7]. The study suffered from a high screen failure rate with the CRO’s assay (labeled as the Version 1 assay for the rest of the document). Among the 393 serologic positive patients screened for the study, only 123 were tested PCR positive and thus eligible for the study.

Our investigation of the Version 1 assay found the persistent problems of inconsistent and low DNA extraction efficiency, poor precision among the technical replicates of reference *T*. *cruzi* DNA used to establish standard curves of kDNA qPCR assay, and highly variable parasite loads among individual 0.2 ml aliquots of the same clinical PAXgene blood sample. We later discovered that a major cause of the substandard performance was the utilization of PAXgene Blood DNA tubes, because the Version 1 assay was designed for the commonly used method for collecting and processing CD blood, which involves the immediate mixing of freshly collected 10 ml EDTA-blood with 10 ml of 6M guanidine hydrochloride and 0.2 M EDTA buffer (GEB), often followed by 10 minute boiling, resulting in the lysis of *T*. *cruzi* in the 10 ml blood, the release of kDNA from *T*. *cruzi* mitochondria, and the decatenation of minicircle DNA from the complex structure of kDNA. The PAXgene tubes could not lyse the cells and thus caused the variable parasite load estimates among individual 0.2 ml aliquots. In addition, PAXgene blood prevented the Version 1 assay from efficiently and reproducibly isolating *T*. *cruzi* DNA. To accurately evaluate the treatment efficacy outcome of the STOP CHAGAS study, we developed and validated a robust, sensitive and specific assay (labeled as the Version 2 assay for the rest of the document) to detect *T*. *cruzi* DNA in PAXgene blood specimens collected from the patients randomized in the study.

## Materials and Methods

### Reference *T*. *cruzi* DNA

Reference DNA of both K98 (a representative strain of *T*. *cruzi* discrete typing unit (DTU) TcI) and CL Brener (TcVI) were obtained from Dr. Alejandro Gabriel Schijman’s laboratory at INGEBI-CONICET, Buenos Aires, Argentina. The stock concentration was 1.5 ng/μl, equivalent to 7.5 x 10^6^ parasites per ml of blood (PPM). Serial titrations were made with low EDTA TE buffer (Cat. Num. 12090–015, Thermo Fisher Scientific, Waltham, MA) containing 10 ng/μl carrier RNA (Cat. Num. 4382878, Thermo Fisher Scientific) so that the DNA dilutions could be stably stored at 4^°^C for at least 1 month.

### Internal Amplification Control (IAC) DNA

A linearized pZErO plasmid containing a sequence of *Arabidopsis thaliana* was also obtained from Dr. Schijman’s laboratory. The stock concentration was 0.6 ng/μl and serial titrations were made with the above mentioned low EDTA TE buffer containing carrier RNA. To evaluate DNA extraction efficiency and to detect potential PCR inhibition from inhibitors such as heme from whole blood, 5 μl of 0.03 pg/μl of IAC DNA was spiked into 200 μl equivalent of clinical PAXgene blood specimens as the internal control. The amount of IAC DNA was chosen so that its corresponding PCR Ct values (around 27) in eluted DNA samples were near the midpoints of kDNA qPCR standard curves for both *T*. *cruzi* reference strains.

### *T*. *cruzi* Negative PAXgene Blood Samples Spiked with IAC and Reference *T*. *cruzi* DNA

20 ml of whole blood specimens per donor were collected in PAXgene Blood DNA tubes (Qiagen, Hilden, Germany) from six normal healthy volunteers (NHVs) from USA-based donors that participated in the company’s Volunteer Donor Program. The routine blood collection procedures were performed by the company’s phlebotomist after the donors signed the research subject information and consent form that was reviewed and approved by Western Institutional Review Board (WIRB). The blood samples were tested negative with *T*. *cruzi* kDNA PCR assay. 5 μl of *T*. *cruzi* DNA with known concentrations spiked into 200 μl of NHVs’ PAXgene blood specimens were used to select and to optimize *T*. *cruzi* DNA isolation methods, and to establish the assay’s sensitivity. NHV PAXgene blood samples spiked with two low concentrations of *T*. *cruzi* DNA, and the ones spiked with only IAC, were utilized as the controls for batched DNA extraction of clinical samples.

### Cell Lysis of PAXgene Blood Specimens and Extraction of *T*. *cruzi* DNA

To lyse *T*. *cruzi*, 5 ml of PAXgene blood was mixed with 5 ml of Genomic Lysis Buffer (Zymo Research, Irvine, CA), followed by 10-minute room temperature incubation. The lysed blood specimens were stored at -80^°^C until DNA extraction. For DNA isolation, 5 μl of 0.03 pg/μl of IAC DNA and 0.6 ml of Genomic Lysis Buffer were added to 0.4 ml of lysed blood sample (equivalent to 0.2 ml of PAXgene blood), mixed and followed by 10 minute room temperature incubation. DNA extraction was performed with Quick-gDNA Blood MiniPrep kit (Zymo Research) per the manufacturer’s instruction except for the DNA elution step, in which 50 μl of heated low EDTA TE buffer (pre-warmed at 70^°^C) was added to the column and incubated at room temperature for 5 minutes before centrifugation to collect extracted DNA.

### Real Time PCR Assay for IAC DNA

The PCR reaction mix consists of 1x TaqMan Universal Master Mix II with UNG (Thermo Fisher Scientific, abbreviated as TaqMan Master Mix), 0.5 μM of each PCR primer, with the forward primer being CCGTCATGGAACAGCACGTA and the reverse primer ACCTTCAAGAACAGATGCTCCAA, and 0.2 μM of FAM labeled TaqMan MGB probe TTGCTGGAGAAATGACT. The PCR amplicon size is 77 base pair. The qPCR plate was prepared with Biomek NX^p^ Laboratory Automation Workstation (Beckman Coulter, Brea, California) so that each sample would have 4 technical replicates per PCR run. We used ViiA^TM^ 7 Real-Time PCR System (Thermo Fisher Scientific), with total qPCR reaction volume at 10 μl including 2 μl of extracted DNA. PCR reaction was programed at 50^°^C for 2 minutes, 95^°^C for 10 minutes, followed by 45 cycles of 95^°^C for 15 seconds and 60^°^C for 1 minute. For analysis settings of ViiA7 Software v1.2.1, we used 0.1 as the threshold and selected automatic baseline determination.

### Real Time PCR Assay for Minicircle DNA of *T*. *cruzi* kDNA

*T*. *cruzi* kDNA qPCR assay targeted the conserved 120-bp repetitive sequences of minicircle DNA. The PCR reaction mix consists of 1x TaqMan Master Mix, 0.9 μM of each PCR primer, with the published forward primer being TTTGGGAGGGGCGTTCA and the published reverse primer ATATTACACCAACCCCAATCGAA[7], and 0.2 μM of newly designed FAM labeled TaqMan MGB probe CCCCCGTACATTATTT. The PCR amplicon size is 117 base pair. The qPCR plate setup and the reaction conditions were the same as for the IAC qPCR assay.

### Establishing Limit of Detection (LOD) of *T*. *cruzi* qPCR Assay

We used serially diluted reference *T*. *cruzi* DNA (CL-Brener or K98) spiked in NHV PAXgene blood to establish analytical sensitivity in terms of LOD. According to the CLSI EP17-A2 guideline [8], LOD is defined as the lowest concentration that at least 95% of the technical replicates have positive signals of the assay. We first searched for the lowest concentrations in which 3 of the 3 technical replicates were tested positive. We then created 21 technical replicates at these lowest concentrations for each of two references and checked if at least 20 out of the 21 replicates were tested positive.

### Establishing *T*. *cruzi* DNA qPCR Amplification Standard Curve Formulas

We investigated two methods of establishing *T*. *cruzi* qPCR standard curve formulas and found both methods produced highly similar results. The first method used serially diluted *T*. *cruzi* DNA spiked-in NHV PAXgene blood samples and involved DNA extraction and qPCR assays to establish an empirical standard curve for each individual run. The second method used an outlier rejection based algorithm. First, for each *T*. *cruzi* DNA dilution, we performed dozens of PCR reactions, with quad-replicates per reaction. Then for each individual reaction’s quad-replicates, we removed the replicate that was most different from the rest of the replicates and calculated the median Ct of the remaining triplicates. Finally, the median of the calculated median Cts was selected to represent the Ct at each *T*. *cruzi* DNA dilution to obtain the respective qPCR standard curve formulas for CL-Brener or K98. We selected the formulas obtained from the second method for analyzing the clinical samples because it could be implemented efficiently and was less prone to run-to-run variability and operator errors.

## Results

### Rational for Re-designing *T*. *cruzi* PCR Assay to Evaluate Treatment Effect of the STOP CHAGAS Study

Although the STOP CHAGAS study used the Version 1 PCR assay for patient enrollment screening, our investigation found that 75% of the clinical samples that were analyzed with the Version 1 assay failed at least one of the qPCR assay QC parameters established by the CRO (S1 Table). The QC parameters (such as PCR amplification efficiency, R^2^ value, and minimum number of data points required for the standard curve) were set generously for the Version 1 kDNA qPCR assay, mainly due to the challenge of establishing reproducible standard curves with reference *T*. *cruzi* DNA (see the solution to the challenge in the section called “Standard Curves of kDNA qPCR Assays”). In order for the clinical trial to accurately evaluate the treatment response, we decided to develop a more sensitive and reproducible qPCR assay accompanied with more stringent quality control and quality assurance parameters to analyze all the clinical samples collected from the enrolled patients including the ones already evaluated by the Version 1 PCR assay.

### Development of *T*. *cruzi* DNA Extraction Procedure for PAXgene Blood Specimens

Although the QIAamp Blood DNA Mini kit (Qiagen) used in Version 1 assay had consistent yields of human genomic DNA (gDNA), and had good RNase P qPCR results indicating integrity of gDNA, it had inconsistent (up to 5 fold differences) and poor (most below 50%) DNA extraction efficiency with IAC DNA, and even lower efficiency with *T*. *cruzi* DNA. We explored various experimental conditions for all major steps of the QIAamp kit and failed to improve the recovery of IAC and *T*. *cruzi* DNA.

**Fig 1:**
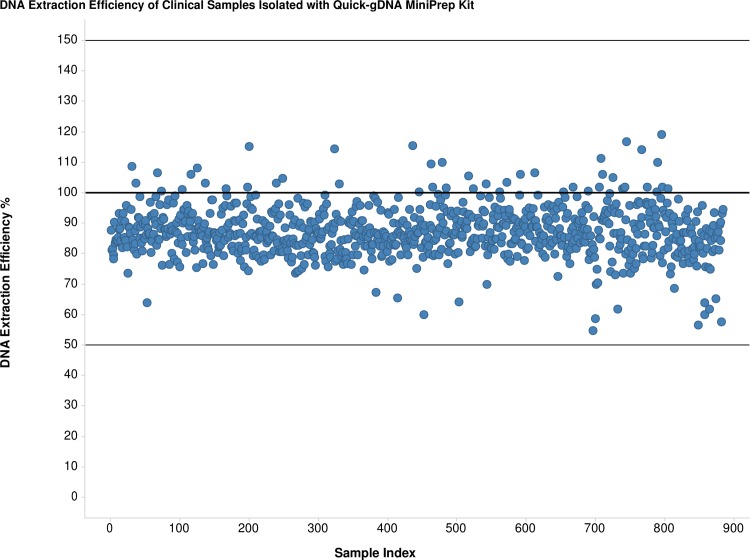
Consistent and High DNA Extraction Efficiency of Quick-gDNA MiniPrep Kit with Clinical PAXgene Blood Samples. The 50% and 150% lines are the lower and upper limits of acceptable values for DNA extraction efficiency.

We found the solution in the Quick-gDNA Blood MiniPrep kit (abbreviated as the ZR kit) that used a simple procedure for quick isolation of total DNA, including gDNA, mitochondrial and viral DNA, without commonly used protease K or organic denaturants. DNA extraction efficiency with IAC DNA spiked in NHVs’ PAXgene blood samples was high and consistent, with an average of 93% with a CV of 7% in eleven independent DNA extraction runs with a total of 144 samples. Fig 1 shows the consistent and high DNA extraction efficiency of the ZR kit with the STOP CHAGAS study’s PAXgene blood samples, with an average of 89% and a CV of 9%. Using one-fourth the elution volume of the QIAamp kit, the ZR kit further improved assay sensitivity through concentrating DNA eluent.

### Lysis of *T*. *cruzi* in PAXgene Blood before DNA Extraction

**Fig 2:**
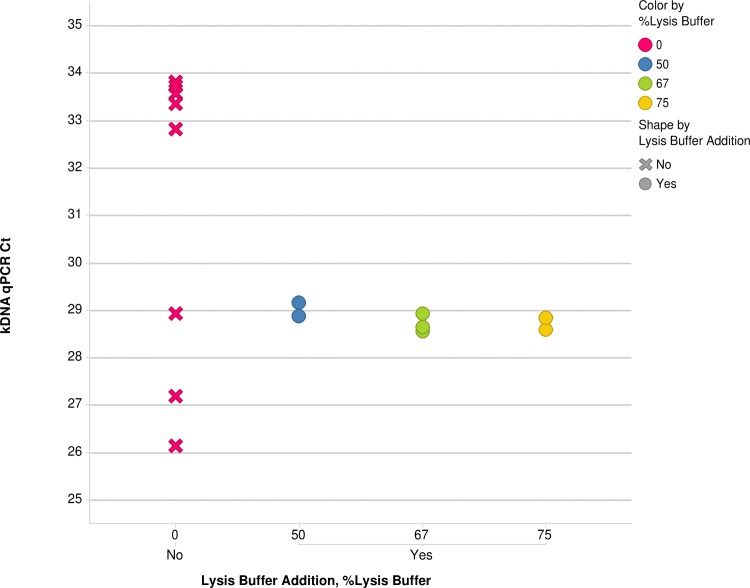
Effect of Addition of Genomic Lysis Buffer to PAXgene Blood on *T*. *cruzi* kDNA qPCR Ct Variability. Increasing percentage of Genomic Lysis Buffer was added to 5 ml of PAXgene blood. 50% v/v of lysis buffer was sufficient to reduce Ct variability caused by sampling error of individual 0.2ml aliquots of un-lysed PAXgene blood.

Based on the good recovery of *T*. *cruzi* DNA spiked in NHV PAXgene blood, we hypothesized that Genomic Lysis Buffer in the ZR kit could efficiently lyse *T*. *cruzi* and to release minicircle DNA in clinical PAXgene blood. Fig 2 shows the results that confirmed the hypothesis. Individual 0.2 ml aliquots of a clinical PAXgene blood sample without the lysis buffer had Ct values ranging from 26.1 to 33.8 for the kDNA qPCR assay, while the addition of increasing amounts of lysis buffer to 5 ml PAXgene blood aliquots resulted in consistent Ct values ranging from 28.5 to 29.2 in 0.2 ml equivalent of PAXgene blood aliquots. To avoid sampling error caused by small aliquots of un-lysed blood, we performed *T*. *cruzi* lysis with 5 ml of the PAXgene blood before DNA extraction.

### Improving TaqMan Based *T*. *cruzi* kDNA qPCR Assay

**Fig 3:**
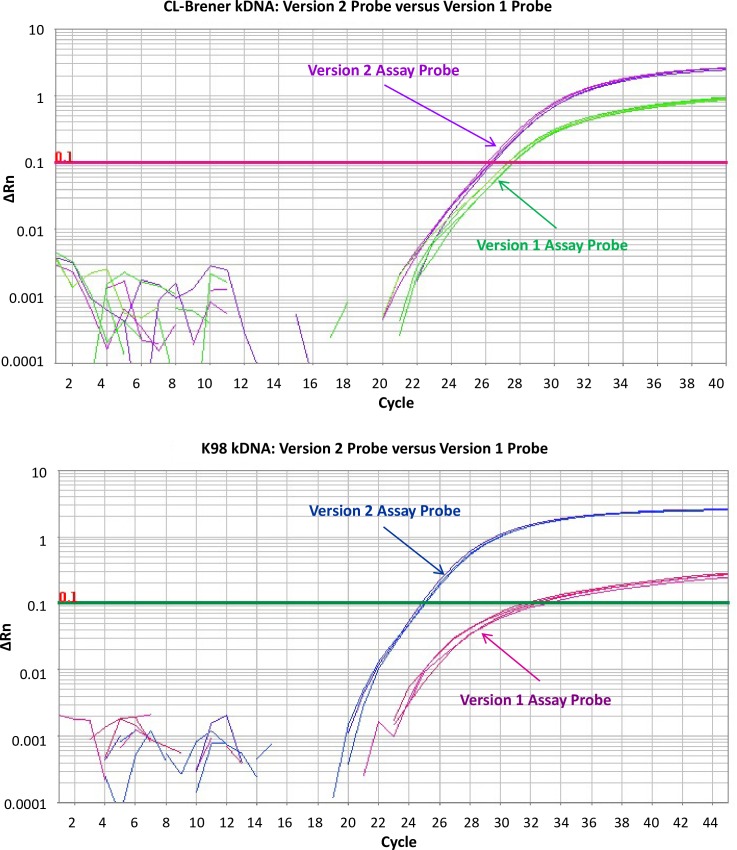
Improving *T*. *cruzi* kDNA qPCR Assay Sensitivity with Version 2 Assay TaqMan Probe. Fig 3A: Comparison of Version 1 and Version 2 probe with 20 fg/μl of CL Brener; Fig 3B: 20 fg/μl of K98 (each DNA sample having four qPCR technical replicates)

We compared Platinum Quantitative PCR SuperMix-UDG w/ROX (Thermo Fisher Scientific, abbreviated as Platinum SuperMix) used in Version 1 assay with TaqMan Master Mix used in Version 2 for both IAC and kDNA qPCR assays. S1A Fig and S2A Fig show that the Version 2 assay’s TaqMan Master Mix had better performance by lowering Ct values and increasing the exponential phases for both qPCR assays. We also re-designed the TaqMan probe, because we found two potential problems with the published probe sequence 5’-CATCTCACCCGTACATT-3′ for MGB probe-based TaqMan assays. First, there is a polymorphism at the seventh nucleotide (“A” or “C”) according to *T*. *cruzi* kinetoplast minicircle sequences in NCBI database, with “C” not “A” shown in the vast majority of *T*. *cruzi* strains. Second the original probe sequence is not optimal for MGB probe based TaqMan assay, because it was designed as a LNA probe. We removed the first five nucleotides of the published probe sequence so that the polymorphic nucleotide “C” was place at the 2nd position instead of being close to the center of the probe, thus having less influence on potential discrimination of sequences with the “A” polymorphism. We added four highly conserved nucleotides at the end for the new probe, so that the new probe could meet the design guidelines of an optimal MGB probe. The newly designed probe resulted in up to 1 Ct improvement for CL Brener (Fig 3A) and up to 7 Ct improvement for K98 (Fig 3B), further increasing the assay’s sensitivity.

### LOD of *T*. *cruzi* qPCR Assay

We used serially diluted reference *T*. *cruzi* DNA spiked in NHV PAXgene blood to establish analytical sensitivity in terms of LOD. Please note that for each technical replicate used to estimate LOD, the extracted DNA was analyzed by three separate runs of kDNA qPCR, with 4 PCR replicates per qPCR run. Only the technical replicates with at least two Ct values less than 45 among the total of twelve Ct results were called positive for the qualitative kDNA PCR assay. One major reason why at least 2 positive PCR amplifications among the 12 qPCR replicates was called positive in Version 2 assay was to be consistent with Version 1 assay in which at least one replicate had to be positive among the 6 qPCR replicates. Table 1 shows that the Version 2 assay has up to 39 fold increase in sensitivity compared to the Version 1 assay.

**Table 1:** Improvement in Sensitivity of *T*. *cruzi* qPCR Assay in Terms of LOD

*T*. *cruzi* DTU	Representative Strain	LOD of Version 1 Assay*	LOD of Version 2 Assay**	Increase in Assay Sensitivity
**I**	K98	1 PPM (0.2 fg/μl)	0.025 PPM (0.005 fg/μl)	39x
**VI**	CL Brener	1 PPM (0.2 fg/μl)	0.05 PPM (0.01 fg/μl)	19x

* Version 1 Assay: LOD established by CRO; Used in Patient Enrollment Screening of the STOP CHAGAS Study

** Version 2 Assay: Used in Evaluating Treatment Response of the STOP CHAGAS Study

### Standard Curves of *T. cruzi* kDNA qPCR Assays

One of the STOP CHAGAS study’s secondary objectives was to evaluate if the response to treatment could be measured by changes in *T*. *cruzi* parasite loads with quantitative PCR. To quantify *T*. *cruzi* parasite loads, the CRO of Version 1 assay established two qPCR standard curves so that K98 representing TcI could be utilized to represent the clinical samples collected from the Northern countries of South America such as Mexico and Columbia, and CL-Brener representing TcVI for the Southern Cone region of South America such as Argentina and Chile. Despite of the better performance of the Version 2 assay, we observed the same phenomenon as seen by the CRO that reference *T*. *cruzi* DNA did not behave as other DNA material, such as gDNA, plasmid DNA, or PCR DNA. S2 Fig shows an example of such atypical behavior, in which the lower concentration *T*. *cruzi* DNA dilution (10 PPM) had less variability in Ct values among the quad-replicates per sample than the higher concentration dilution (100 PPM), in which one of the four replicates tended to have much lower Ct values than the remaining three replicates that usually had consistent Ct values. We also observed another atypical behavior of reference *T*. *cruzi* DNA in which the Ct difference between the different 10 fold dilutions could be different from the expected 3.32 value. We suspected that the atypical behaviors might be caused by the complex *T*. *cruzi* kDNA structure that is composed of thousands of interlocked minicircle DNAs and dozens of maxicircle DNAs. We hypothesize some of the reference *T*. *cruzi* DNA dilutions might still contain the interlocked minicircle DNA targeted by the qPCR assay. We explored many experimental conditions to decatenate kDNA while avoiding DNA degradation, but failed to find one that could reduce technical qPCR variability for all dilutions while maintaining the 3.32 Ct difference expected between the 10-fold DNA dilutions.

**Fig 4:**
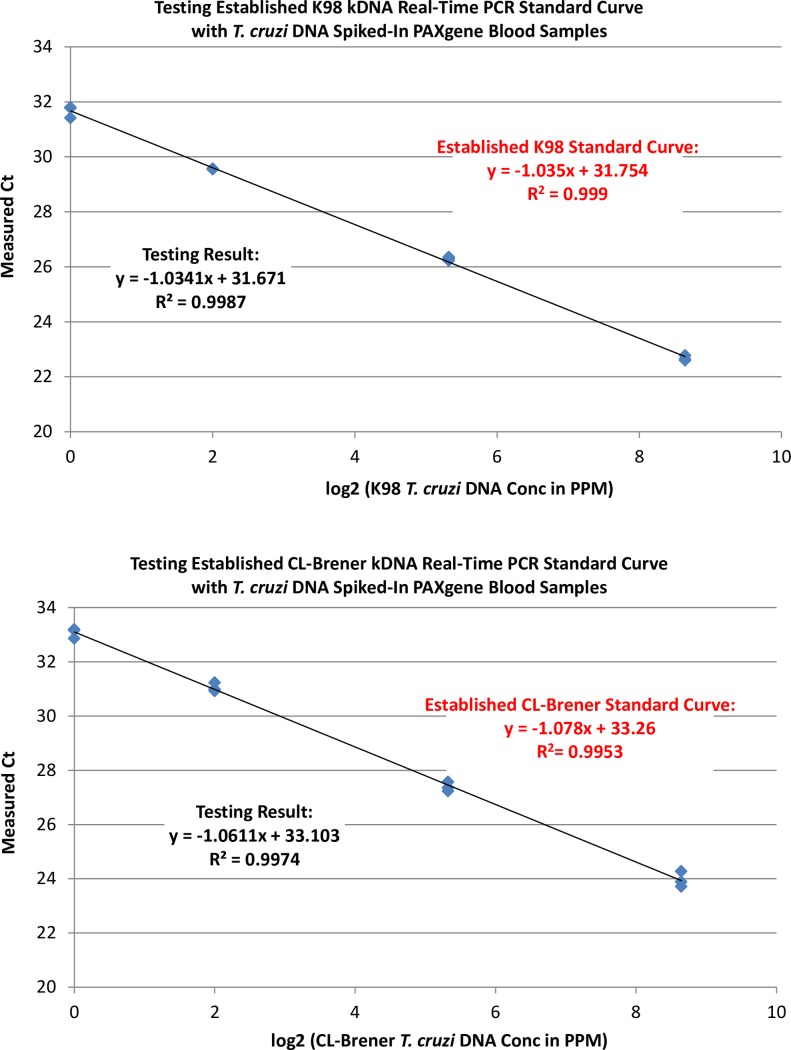
Confirming Accuracy of Established *T*. *cruzi* kDNA qPCR Standard Curves with NHV PAXgene Blood Samples Spiked with Known Concentrations of *T*. *cruzi* DNA. Fig 4A: CL Brener; Fig 4B: K98; with 3 PAXgene blood samples per concentration per *T*. *cruzi* strain

Because the observed Ct variability of *T*. *cruzi* DNA dilutions seemed to be caused by the unpredictable drop of Ct in one technical replicate (the outlier replicate), while the overall median Cts maintained the expected Cts, we decided to establish kDNA qPCR standard curves for both strains with an outlier rejection based algorithm. First, we obtained dozens of quad-replicate Ct values at each *T*. *cruzi* DNA dilution. Then for each quad-replicate, we removed the outlier replicate and calculated the median Ct of the remaining triplicates. Finally, the median of the calculated median Cts was selected to represent the Ct at each *T*. *cruzi* DNA dilution. Table 2 shows the formulas of the established qPCR standard curves for the two reference strains. To check the accuracy of the established standard curves, we measured the Ct values of *T*. *cruzi* DNA extracted from the NHV PAXgene blood samples spiked with known concentration *T*. *cruzi* DNA. Fig 4 shows the almost identical formulas and R^2^ between the established standard curves and the measured empirical ones for both references, demonstrating the accuracy of the established qPCR standard curves.

**Table 2:** Established *T*. *cruzi* kDNA qPCR Standard Curve Formulas of the Version 2 Assay

T. cruzi Strain	Formula with “y” in Ct unit	PCR Amplification Efficiency	R^2^
**K98**	y = -1.078 x log2(PPM) + 33.26	95.3%	0.999
**CL Brener**	y = -1.035 x log2(PPM) + 31.75	90.2%	0.995

### Accuracy and Precision of *T*. *cruzi* kDNA qPCR Assay

To examine the accuracy and precision of the qPCR assay, we spiked in 100, 10, 1, and 0.25 PPM equivalent *T*. *cruzi* DNA of CL Brener or K98 into 3 NHVs’ PAXgene blood specimens. The spiked-in PAXgene blood specimens were extracted on different days, and on each day at least 3 intra-run extractions were performed for each PAXgene blood sample. Table 3 summarizes the results presented in S3 Fig, which shows that the measured *T*. *cruzi* parasite loads were close to the expected values, and the measured values were consistent within the same run and among different runs for both *T*. *cruzi* strains.

**Table 3:** Accuracy and Precision of Version 2 *T*. *cruzi* kDNA qPCR Assay

*T*. *cruzi* Strain	Spike-in *T*. *cruzi* DNA Concentration (PPM*)	Measured *T*. *cruzi* DNA Concentration (PPM*)	CV% of Measured *T*. *cruzi* DNA Concentration
**K98**	100	124.2	5.9
**K98**	10	10.3	8.4
**K98**	1	1.00	23.8
**K98**	0.25	0.19	22.6
**CL Brener**	100	141.0	14.1
**CL Brener**	10	12.3	22.6
**CL Brener**	1	0.98	26.6
**CL Brener**	0.25	0.19	41.7

### Quality Controls for qPCR Analysis of Clinical Samples of the STOP CHAGAS Study

Table 4 lists the multiple controls for both DNA extraction and qPCR to ensure data quality. For DNA extraction, two types of controls were implemented. One was IAC DNA added to each clinical PAXgene blood samples to measure DNA extraction efficiency and PCR inhibition. The other was a set of three controls of spiked-in NHV PAXgene blood samples that were extracted along with each batch of clinical samples. For the qPCR reactions, in addition to the samples extracted from the DNA extraction controls, three individual negative controls of NTC (low EDTA TE buffer with carrier RNA) and one positive control of 10 PPM *T*. *cruzi* DNA were analyzed for each PCR run. Table 4 shows all clinical samples from patients enrolled in the STOP CHAGAS study passed the QC parameters established for the assay.

**Table 4:** QC Parameters, Acceptable Values, and Final Results of Analysis of Clinical Samples Collected from the Randomized Patients of the STOP CHAGAS Study

Experimental Steps	QC Parameters	Acceptable Values	Results from Clinical Sample Analysis
**DNA Extraction**	Negative PAXgene Blood Control	≤1 detectable Ct value in all 12 replicates of kDNA qPCR assay	All runs had no detectable Ct and passed.
**DNA Extraction**	DNA Extraction Efficiency	50 to 150%	All samples passed.
**DNA Extraction**	DNA Extraction of 0.25 PPM Positive PAXgene Blood Control	Within each of the 3 quad-replicates, at least 2 replicates had detectable Ct.	For K98, all runs had 100% detectable Cts in quad-replicates and passed.
** **			For CL Brener, 6% of the runs had 3 detectable Cts in quad-replicates, and the rest had all 4 detectable Cts. All runs passed.
**DNA Extraction**	DNA Extraction of 1 PPM Positive PAXgene Blood Control	Within each of the 3 quad-replicates of kDNA PCR assay, at least 3 replicates had detectable Ct.	For K98, all runs had all 4 detectable Cts in quad-replicates. All runs passed.
** **			For CL Brener, 2% of the runs with 3 detectable Cts in quad-replicates, the rest all 4 detectable Cts. All runs passed.
**qPCR**	10 PPM DNA Positive Control	For K98: mean Ct between 27.99 and 29.27	All runs passed.
** **		For CL Brener: mean Ct between 29.43 and 30.68	All runs passed.
**qPCR**	NTC Negative Control	≤ 1 detectable Ct value in all 12 replicates of kDNA qPCR	All runs had no detectable Ct and passed.

Please note that for negative controls, we utilized “≤ 1” instead of “<1” as acceptance values, even though we did not see any amplification (Ct < 45) in negative control samples during clinical analysis. However, the acceptance values were established during assay validation, in which a couple of negative samples had 1 amplification with Ct > 35 among the 12 replicates.

## Discussion

Although there are a number of qPCR methods [7, 9, 10] that had been published for detecting circulating *T*. *cruzi*, our method was the first to detect *T*. *cruzi* in whole blood specimens collected with PAXgene Blood DNA tubes, which had the benefits of reduced cost and the potential to reduce clinical site-specific artifacts caused by the more complicated collection and processing procedures for multicenter global clinical trials. However, due to the complex structure of *T*. *cruzi* DNA, previously published methods were not adequate for detecting *T*. *cruzi* in PAXgene blood. Our PCR assay overcame the inadequacy by finding simple methods that efficiently extract *T*. *cruzi* DNA, and by improving sensitivity of the kDNA qPCR assay. We also solved the problem of unreliable kDNA qPCR standard curves caused by kDNA structure through a statistical algorithm. The established standard curve was essentially identical to the empirical ones obtained with NHV PAXgene blood spiked with known concentrations of *T*. *cruzi* DNA, but was much easier to implement than to build empirical standard curves for each individual qPCR run.

We compared the LOD of our *T*. *cruzi* qPCR assays with other published methods and find that our assay had similar or higher sensitivity compared to some published sensitive PCR methods [7, 11]. The high sensitivity of Version 2 assay is likely due to both the optimized assay conditions and the stricter definition of the negative calls of the qualitative PCR assay. In one similarly designed randomized clinical trial comparing POS to BNZ in treating chronic CD [12], a sample could be called negative if it had four positive amplifications, all with Ct between 40 and 45, among the four PCR replicates. Such sample would be called positive in our definition. By lowering the chance of false negative calls, we reduced the likelihood of false positives of treatment success responses.

We did not formally establish the limit of quantitation (LOQ) during assay validation. Instead, we used the following three strict criteria to evaluate the reliability of the quantitative results. First, the four technical replicates per qPCR run must pass an in-house established variability test before their mean Ct was used to calculate parasite load. If any of the three kDNA qPCR runs failed the Ct variability rules, the results would be labeled as unreliable. Second, if any of the three runs had a mean Ct above 35, the data were also called unreliable. Third, if CV% of the parasitic loads of the three kDNA qPCR assays runs was greater than 30%, the results were also labeled as unreliable. For CL-Brener DNA, we observed that when samples had at least 0.05 fg/μl of DNA, 95% of them would pass all three criteria. However, for samples with less than 0.05 fg/μl of DNA, 94% of them would fail at least one of the three criteria. Therefore, we estimate that the LOQ of the assay to detect CL-Brener was around 0.05 fg/μl. Although we did not have sufficient K98 data points to estimate its empirical LOQ, because K98 has better LOD than CL-Brener, we estimated that both K98 and CL-Brener would have LOQ around 0.05 fg/μl, equivalent to 0.25 PPM, similar to the 0.90 PPM LOQ of a published assay[11].

We learned a few lessons during the assay development. We found that good RNase P assay results of human gDNA [11] were not informative in selecting *T*. *cruzi* DNA extraction method due to the difference between the two DNA. NHV PAXgene blood spiked with known concentrations of *T*. *cruzi* DNA was a better approach to evaluate *T*. *cruzi* DNA extraction. To avoid false negative PCR results caused by the sampling error of small aliquots of un-lysed blood specimens, it is important to perform *T*. *cruzi* lysis with a sufficient amount of blood (5–10 ml) before DNA extraction. The commonly utilized method of mixing 10 ml of EDTA-blood with 10 ml of GEB alleviates such sampling error, while other published methods such as mixing only 0.2 ml of EDTA-blood with 0.2 ml of GEB or directly extracting *T*. *cruzi* DNA from 0.2 ml of EDTA-blood would likely suffer from the sampling error. Avoiding such sample error is especially critical for samples collected from asymptomatic CD patients due to low levels of circulating *T*. *cruzi*.

## Conclusions

In the manuscript detailing the clinical results of the STOP CHAGAS study [13] that utilized our assay, one of the major conclusions is the strong support of the use of the *T*. *cruzi* qPCR assay as a marker of therapeutic response in subjects with chronic asymptomatic CD. In addition to the excellent sensitivity, specificity and precision, our assay allows the simple and cost-effective PAXgene tubes to collect blood specimens from CD patients.

## Supporting Information

S1 Table QC Parameters and Acceptable Values Set for the Version 1 PCR Assay (DOCX)

S1 Fig Improving TaqMan qPCR Assay with TaqMan Master Mix Sup Fig 1A: IAC qPCR Assay with 0.15 pg/μl of DNA; Sup Fig 1B: *T*. *cruzi* kDNA qPCR Assay with 20 fg/μl of CL Brener DNA (Each sample with 4 qPCR technical replicates) (TIF)

S2 Fig Atypical qPCR Behavior of Reference *T*. *cruzi* DNA Dilutions–The 10 PPM dilution (in blue color) had less technical variability in Ct values than the 100 PPM dilution (in pink color) even though it had higher mean Ct values. NOTE: Data points on each vertical line present the Ct values of technical quad qPCR replicates of each reference *T*. *cruzi* DNA dilution sample of CL-Brener.
(TIF)

S3 Fig Measured *T*. *cruzi* PPM versus Expected PPM with NHV PAXgene Blood Samples Spiked with Known Concentrations of CL Brener and K98 *T*. *cruzi* DNA Sup Fig 3A: K98 DNA Spiked at Concentrations of 100, 10, 1, and 0.25 PPM in 3 NHVs’ PAXgene Blood Samples; Sup Fig 3B: CL Brener DNA Spiked in PAXgene Samples; with DNA Isolated and Analyzed in Multiple Days, with at Least 3 Intra-run Replicates per Day (TIF)
