# Supplementary material for: Development of a PCR Assay to Detect Low Level Trypanosoma cruzi in Blood Specimens Collected with PAXgene Blood DNA Tubes for Clinical Trials Treating Chagas Disease
Source: PLoS Negl Trop Dis. 2016 Dec 1;10(12):e0005146. doi: 10.1371/journal.pntd.0005146 (PMC5131911; doi:10.1371/journal.pntd.0005146)
Supplement: S1 Table — (DOCX) [file pntd.0005146.s001.docx]

**S1 Table: QC Parameters and Acceptable Values Set for the Version 1 PCR Assay**

| **QC Parameters for Version 1 qPCR Assay** | **Acceptable Values** |
| --- | --- |
| DNA Extraction Efficiency (%) | 50 to 200% |
| R^2^ of *T. cruzi* kDNA qPCR Standard Curve | ≥0.85 |
| kDNA qPCR Standard Curve Amplification Efficiency | 70 to 115% |
| Maximum Percentage of Data Points Allowed to Be Discarded for kDNA qPCR Standard Curve | 30% |
| R^2^ of IAC qPCR Standard Curve | ≥0.98 |
| IAC qPCR Standard Curve Amplification Efficiency | 90-110% |
| Maximum Percentage of Data Points Allowed to be Discarded for IAC qPCR Standard Curve | 20% |
