# Supplementary figures and images for: Development of a PCR Assay to Detect Low Level Trypanosoma cruzi in Blood Specimens Collected with PAXgene Blood DNA Tubes for Clinical Trials Treating Chagas Disease
Source: PLoS Negl Trop Dis. 2016 Dec 1;10(12):e0005146. doi: 10.1371/journal.pntd.0005146 (PMC5131911; doi:10.1371/journal.pntd.0005146)

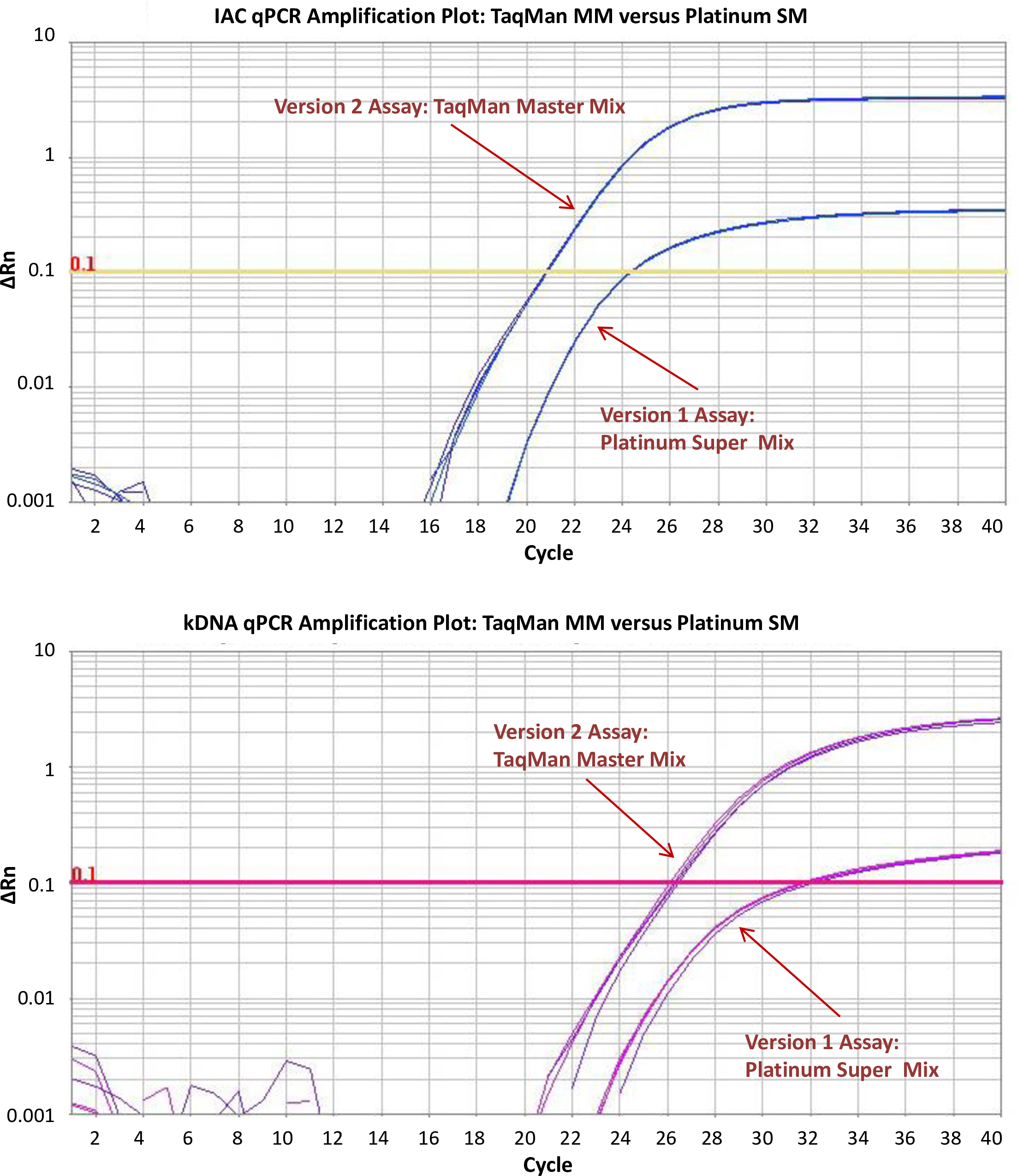

Supplement: S1 Fig — (TIF) [file pntd.0005146.s002.tif]

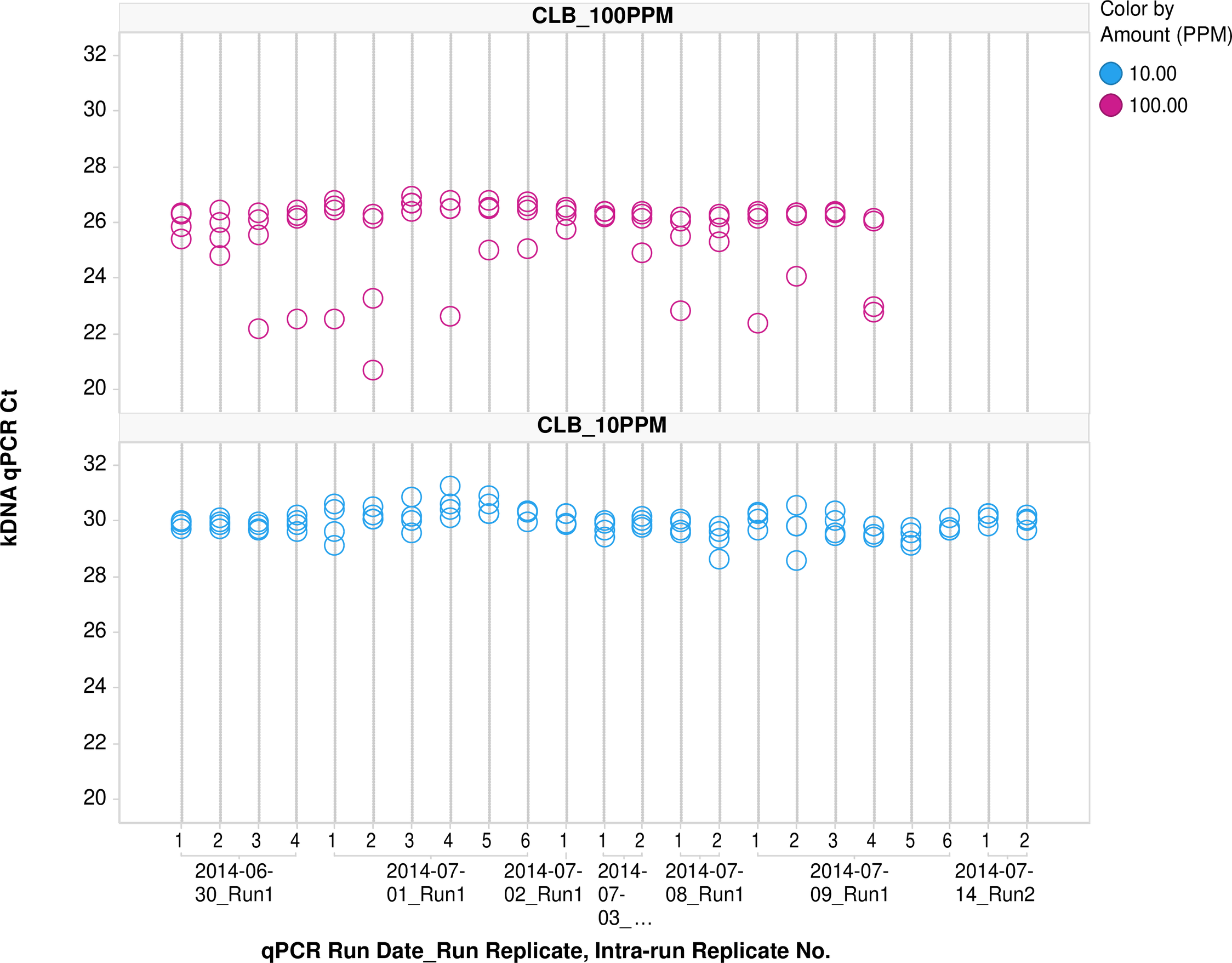

Supplement: S2 Fig — NOTE: Data points on each vertical line present the Ct values of technical quad qPCR replicates of each reference T. cruzi DNA dilution sample of CL-Brener. (TIF) [file pntd.0005146.s003.tif]

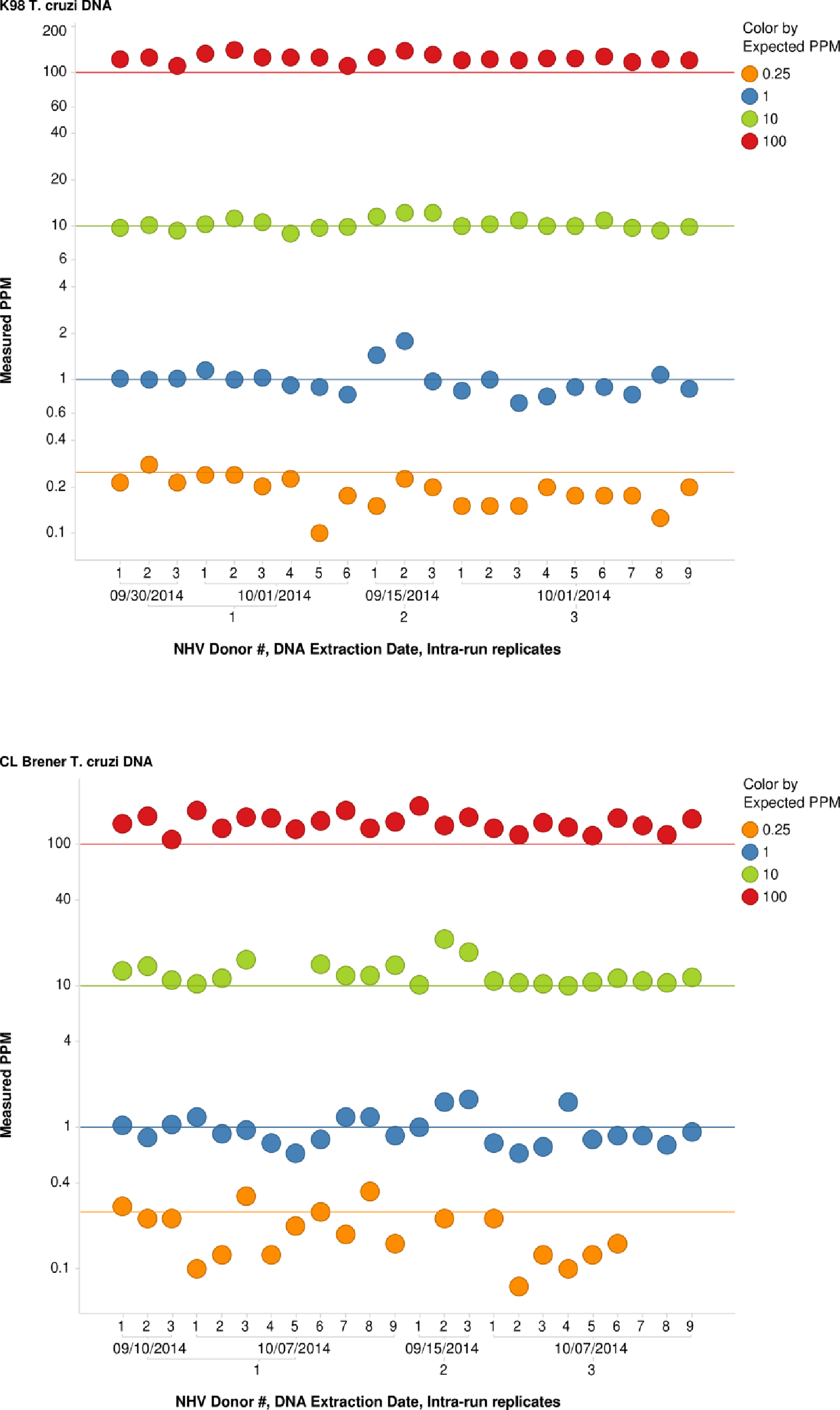

Supplement: S3 Fig — (TIF) [file pntd.0005146.s004.tif]
